# Supplementary material for: Comparative genomics provides new insights into the diversity, physiology, and sexuality of the only industrially exploited tremellomycete: Phaffia rhodozyma
Source: BMC Genomics. 2016 Nov 9;17:901. doi: 10.1186/s12864-016-3244-7 (PMC5103461; doi:10.1186/s12864-016-3244-7)
Supplement: Additional file 6: — List of orphan genes with links to PFAM (related to Additional file 1: Table S1). (ZIP 1428 kb) [file 12864_2016_3244_MOESM6_ESM.zip › BLAST_HTML_FTR/G04008_P.html]

BLAST Search Results


```
BLASTP 2.2.27+


Reference:
Stephen F. Altschul, Thomas L. Madden, Alejandro A. Schäffer,
Jinghui Zhang, Zheng Zhang, Webb Miller, and David J. Lipman (1997),
"Gapped BLAST and PSI-BLAST: a new generation of protein database
search programs", Nucleic Acids Res. 25:3389-3402.


Reference for
composition-based statistics:
Alejandro A. Schäffer, L. Aravind, Thomas L. Madden, Sergei
Shavirin, John L. Spouge, Yuri I. Wolf, Eugene V. Koonin, and
Stephen F. Altschul (2001), "Improving the accuracy of PSI-BLAST
protein database searches with composition-based statistics and
other refinements", Nucleic Acids Res. 29:2994-3005.


Database: nr
           71,551,133 sequences; 26,053,659,533 total letters


Query= G04008_P

Length=880
                                                                      Score     E
Sequences producing significant alignments:                          (Bits)  Value

emb|CED85072.1|  hypothetical protein [Xanthophyllomyces dendrorh...  1315    0.0  


 >emb|CED85072.1| hypothetical protein [Xanthophyllomyces dendrorhous]
Length=794

 Score = 1315 bits (3404),  Expect = 0.0, Method: Compositional matrix adjust.
 Identities = 794/794 (100%), Positives = 794/794 (100%), Gaps = 0/794 (0%)

Query  86   MYPELPPPSYHPICPSCLPSVSLPPISPSPPPPLTLSLSYTHVPPTRPPICITPPLPPLP  145
            MYPELPPPSYHPICPSCLPSVSLPPISPSPPPPLTLSLSYTHVPPTRPPICITPPLPPLP
Sbjct  1    MYPELPPPSYHPICPSCLPSVSLPPISPSPPPPLTLSLSYTHVPPTRPPICITPPLPPLP  60

Query  146  SPPLPPLPALPPPPPTPPFIRGRTHHANTWPSQQQSLPSITHRPQTRALPIIPSGPLVPS  205
            SPPLPPLPALPPPPPTPPFIRGRTHHANTWPSQQQSLPSITHRPQTRALPIIPSGPLVPS
Sbjct  61   SPPLPPLPALPPPPPTPPFIRGRTHHANTWPSQQQSLPSITHRPQTRALPIIPSGPLVPS  120

Query  206  VSDPVPVSLSRPTAESPSASHPIHDSLSIQPDRPRPFDQTADNEDNENNSDIARALKHSL  265
            VSDPVPVSLSRPTAESPSASHPIHDSLSIQPDRPRPFDQTADNEDNENNSDIARALKHSL
Sbjct  121  VSDPVPVSLSRPTAESPSASHPIHDSLSIQPDRPRPFDQTADNEDNENNSDIARALKHSL  180

Query  266  TLSWKVSPPVRKFEDLQASFLTKDGKKSQVSPLAEEYKRLQFQPDQAQRPTASPRQSTKH  325
            TLSWKVSPPVRKFEDLQASFLTKDGKKSQVSPLAEEYKRLQFQPDQAQRPTASPRQSTKH
Sbjct  181  TLSWKVSPPVRKFEDLQASFLTKDGKKSQVSPLAEEYKRLQFQPDQAQRPTASPRQSTKH  240

Query  326  PAIAPLSSSLHHRRHRPPPLPIRPGLPPSHSGPSRLETQQTPVSQAHAPLSLTPAKDTPP  385
            PAIAPLSSSLHHRRHRPPPLPIRPGLPPSHSGPSRLETQQTPVSQAHAPLSLTPAKDTPP
Sbjct  241  PAIAPLSSSLHHRRHRPPPLPIRPGLPPSHSGPSRLETQQTPVSQAHAPLSLTPAKDTPP  300

Query  386  VRPVGPPIQSTGALARTPTLGWARTIISRSLRSDKARVETPGLTSSPPTTEDDRSLDLDG  445
            VRPVGPPIQSTGALARTPTLGWARTIISRSLRSDKARVETPGLTSSPPTTEDDRSLDLDG
Sbjct  301  VRPVGPPIQSTGALARTPTLGWARTIISRSLRSDKARVETPGLTSSPPTTEDDRSLDLDG  360

Query  446  PSERVLRMKPARPILEIDADVIDNDSTRLFPGRLVEGVDAIENSQDEELGRLIYGKTTTS  505
            PSERVLRMKPARPILEIDADVIDNDSTRLFPGRLVEGVDAIENSQDEELGRLIYGKTTTS
Sbjct  361  PSERVLRMKPARPILEIDADVIDNDSTRLFPGRLVEGVDAIENSQDEELGRLIYGKTTTS  420

Query  506  PPTLSPLHAFPQIPVIPIPPVQTGALYIIARTYADLVEELFSIGYVALEGSTDWAFALQI  565
            PPTLSPLHAFPQIPVIPIPPVQTGALYIIARTYADLVEELFSIGYVALEGSTDWAFALQI
Sbjct  421  PPTLSPLHAFPQIPVIPIPPVQTGALYIIARTYADLVEELFSIGYVALEGSTDWAFALQI  480

Query  566  VSIQCGGGGSNDDDDGGKGGLEETEIEDEPDSAIVLWIQDKAKPGERKTRPIPTDQSLTF  625
            VSIQCGGGGSNDDDDGGKGGLEETEIEDEPDSAIVLWIQDKAKPGERKTRPIPTDQSLTF
Sbjct  481  VSIQCGGGGSNDDDDGGKGGLEETEIEDEPDSAIVLWIQDKAKPGERKTRPIPTDQSLTF  540

Query  626  ASPKTRQRKRLIIPRRSRIDQPSSSPLTSSPSFMSPSPSSSSAVSPSLPSPLPLSTSSIS  685
            ASPKTRQRKRLIIPRRSRIDQPSSSPLTSSPSFMSPSPSSSSAVSPSLPSPLPLSTSSIS
Sbjct  541  ASPKTRQRKRLIIPRRSRIDQPSSSPLTSSPSFMSPSPSSSSAVSPSLPSPLPLSTSSIS  600

Query  686  SPLLPIPSRSIESSARLVFQLPGFLPSDRSSVKSTLIPLPSSQAEIEDLTGLVDHLIGLV  745
            SPLLPIPSRSIESSARLVFQLPGFLPSDRSSVKSTLIPLPSSQAEIEDLTGLVDHLIGLV
Sbjct  601  SPLLPIPSRSIESSARLVFQLPGFLPSDRSSVKSTLIPLPSSQAEIEDLTGLVDHLIGLV  660

Query  746  EPWGEFNQSSSGTRPARIPPWRLWTEGEHARFLSMITQSRRRPCLPSGLLPPISTSRTPE  805
            EPWGEFNQSSSGTRPARIPPWRLWTEGEHARFLSMITQSRRRPCLPSGLLPPISTSRTPE
Sbjct  661  EPWGEFNQSSSGTRPARIPPWRLWTEGEHARFLSMITQSRRRPCLPSGLLPPISTSRTPE  720

Query  806  AKAEAGRMEAQLGDQGDSNKRQRIKTWLGRTRLGSVVGWDGAGAGEGQVVQSTDVLHPAG  865
            AKAEAGRMEAQLGDQGDSNKRQRIKTWLGRTRLGSVVGWDGAGAGEGQVVQSTDVLHPAG
Sbjct  721  AKAEAGRMEAQLGDQGDSNKRQRIKTWLGRTRLGSVVGWDGAGAGEGQVVQSTDVLHPAG  780

Query  866  FITPFTFDRSLSEP  879
            FITPFTFDRSLSEP
Sbjct  781  FITPFTFDRSLSEP  794


Lambda      K        H        a         alpha
   0.313    0.133    0.409    0.792     4.96 

Gapped
Lambda      K        H        a         alpha    sigma
   0.267   0.0410    0.140     1.90     42.6     43.6 

Effective search space used: 10515944342160


  Database: nr
    Posted date:  Sep 23, 2015 12:05 AM
  Number of letters in database: 26,053,659,533
  Number of sequences in database:  71,551,133


Matrix: BLOSUM62
Gap Penalties: Existence: 11, Extension: 1
Neighboring words threshold: 11
Window for multiple hits: 40
```
